# Carnosine supplementation improves cognitive outcomes in younger participants of the NEAT trial

**DOI:** 10.1016/j.neurot.2025.e00541

**Published:** 2025-02-06

**Authors:** Timothy E. O'Toole, Alok R. Amraotkar, Hong Gao, Clara G. Sears, Shesh N. Rai, Mathias Basner, Aruni Bhatnagar

**Affiliations:** a Christina Lee Brown Envirome Institute, University of Louisville, Louisville, KY, USA; b Division of Environmental Medicine, Department of Medicine, University of Louisville, Louisville, KY, USA; c Biostatistics and Informatics Shared Resource, University of Cincinnati Cancer Center; Cancer Data Science Center, Department of Biostatistics, Health Informatics and Data Sciences, University of Cincinnati College of Medicine, Cincinnati, OH, USA; d Unit of Experimental Psychiatry, Division of Sleep and Chronobiology, Department of Psychiatry, University of Pennsylvania Perelman School of Medicine, Philadelphia, PA, USA

**Keywords:** Carnosine, Cognition, Clinical trial

## Abstract

Some prior studies suggested that supplementation with carnosine or β-alanine can improve cognitive abilities and neurodegenerative disorders in certain elderly or at-risk populations. However, the efficacy of carnosine in improving cognitive performance in a healthy, adult population has not been assessed. We examined this as a post-hoc secondary outcome in the placebo-controlled, randomized Nucleophilic Defense Against PM Toxicity (NEAT) clinical trial (NCT03314987). Participants in this trial were instructed to take either cornstarch (placebo) or carnosine capsules (2g daily) for up to 12wk. Cognitive ability was assessed using the *Cognition* test battery, which consists of ten individual tests known to engage specific brain systems and covering a range of cognitive domains. Speed, accuracy, and efficiency were obtained for the whole battery as well as for each of the ten individual tests. Participant testing occurred at baseline, prior to randomization, after approximately 6wk of supplementation (Follow-up-1), and after approximately 12wk of supplementation (Follow-up-2). Of the 299 participants who were randomized, we obtained useable measures for 242 participants at Follow-up-1 and 231 ​at Follow-up-2. Age-based stratification (23–35 years, 36–50 years, 51–65 years), showed statistically significant improvements in overall speed and efficiency in the youngest age group stratum at both follow-up visits. This same group also demonstrated significant improvements in seven speed or accuracy scores of the individual tests. The other age groups demonstrated few or no significant improvements. Thus, in a study population largely devoid of susceptibility factors or pre-existing conditions, carnosine supplementation selectively improved high-level cognitive performance in young individuals.

## Introduction

The heart, skeletal muscle, and brain contain high levels of carnosine and anserine [[1], [2], [3]]. Although the functions of these histidyl dipeptides remain unclear, evidence suggests that they participate in buffering intracellular pH, can chelate metals, quench singlet oxygen, and bind reactive carbonyls, resulting in strong antioxidant, anti-inflammatory and anti-glycating activities [3,4]. High levels of carnosine are present in almost all regions of the brain [5,6], suggesting functional importance. This importance is also underscored by the fact that individuals with a genetic deficiency in carnosinase, an enzyme required for the hydrolysis and uptake of carnosine, results in progressive neurological disorder [7]. Other studies show that cognitive decline in subjects with probable Alzheimer's disease is associated with decreased levels of plasma carnosine [8]. Moreover, preclinical studies have shown that carnosine supplementation could ameliorate cognitive function in rodent models of aging [9,10], diabetes [11,12], and Alzheimer's disease [13], and relieve hippocampal neurodegeneration in *db/db* mice [14] as well.

Previous clinical trials have shown that supplementation with carnosine, its precursor β-alanine, or anserine can improve neurological symptoms in those with Parkinson's disease [15] and in older adults with mild cognitive impairment [16]. Carnosine supplementation can also reduce negative symptoms associated with schizophrenia [17]. However, the assessment of histidyl dipeptide-induced changes in cognitive function has yielded mixed results [18]. Some studies have reported improvements in verbal episodic memory, but little or no change in other measures of cognitive function [19]. These assessments however, were derived from small trials (<60 participants) and limited to elderly individuals testing for memory deficits and cognitive decline [18,19]. Most of the positive results have been limited to those with low cognitive assessment scores [20,21].

Therefore, to examine the more general role of carnosine in physiology, rather than pathology, we examined the effects of supplementation on cognitive function in a post-hoc, secondary outcome analysis of the larger double-blind, placebo controlled Nucleophilic Defense Against PM Toxicity (NEAT) clinical trial (NCT03314987) [22]. We did this using the *Cognition* test battery [23], which measures high-level cognitive performance, rather than other assessments which are useful for the early detection of mild cognitive impairment, clinical dementia, or suspected memory deficits. Using data from this longitudinal clinical trial [22], we tested the hypothesis that supplementation with carnosine in healthy adults will improve cognitive function as reflected by enhanced executive function, episodic memory, cognition, and sensorimotor speed.

## Materials and methods

### Study cohort

This study used data from participants of the NEAT clinical trial who gave informed consent. A complete description of this trial, including its cohort, sample size calculations, and endpoints has been published [22]. After enrollment and qualification for this study, participants had 3 clinical visits for collection of biospecimens and clinical testing, including that for cognition. These clinical visits consisted of an initial visit (Baseline) and two subsequent follow-up visits after placebo or carnosine (Fig. S1) supplementation (Follow-up-1: 40.3 ​± ​4.6 days of supplementation; Follow-up-2: 77.4 ​± ​5.7 days of supplementation). The supplementation consisted of the consumption of identically appearing placebo (cornstarch) or carnosine capsules (2 ​× ​0.5g, twice per day). This dose and treatment regimen have been widely used in other carnosine supplementation studies [[24], [25], [26], [27]] and have proven efficacy in increasing basal urinary levels [24,25]. Unused capsules counted at Follow-up-1 and Follow-up-2 were used to assess level of compliance. This trial has been carried out in accordance with The Code of Ethics of the World Medical Association and has Institutional approval (IRB #20.0258). This study is registered at ClinicalTrials.gov (NCT03314987).

### Cognitive testing

The validated, computerized cognitive test battery used (*Cognition*) consists of ten brief tests that assess largely orthogonal cognitive domains [23]. The brain regions primarily recruited by each test have been previously established (Table S1). The majority of these tests are based on the widely used and validated Computerized Neurocognitive Battery (CNB) [28]. *Cognition* was administered on laptop computers (Dell Latitude E7250, 12-inch diagonal screen) calibrated for timing accuracy. Participants performed a brief practice version of each test before initial administration. Prior to analysis, test scores were adjusted for practice and stimulus effects [29] and then z-transformed based on the mean and standard deviation of all administrations to allow for comparisons across tests. After transformation, higher test scores correspond to faster or more accurate performance, respectively. Overall speed and accuracy scores across cognitive domains were generated by averaging z-transformed speed and accuracy scores of the ten *Cognition* tests. Finally, an overall efficiency score was generated by averaging the overall speed and accuracy scores. Prior to data analyses, individual scores were inspected and instances of technical issues or participant non-compliance were excluded from data analysis (0.56 ​% of all data).

### Data analysis

To examine the impact of age and sex on overall scores, as well as scores on each of the individual tests, a one-way analysis of variance (ANOVA) was conducted. Three age groups (23–35 years old; 36–50 years old; 51–65years old) were used. A Student's t-test was used to compare the scores between males and females. To examine the effects of carnosine supplementation on the overall cognitive functions, mixed effect models were used with the cognition scores as dependent variables and treatment (carnosine vs. placebo) as the independent variable from Baseline, Follow-up-1 and Follow-up-2. We used two models with progressive degrees of adjustment. Model-1 used the visit number to indicate the repeated measures of cognitive scores with no other adjustment. In Model-2, we further adjusted for age, sex and race. Age was omitted when the mixed effect models were constructed in the age-stratified subgroups. Estimated fixed effect coefficients (β) for the treatment (carnosine vs. placebo, with placebo as the reference) against the overall cognition scores were presented in forest plots. Similar mixed effect models were constructed using speed and accuracy scores of the 10 individual tests as the dependent variables. These models used the total study population and the three stratified age groups. We also tested for interactions between treatment and age by including an interaction term (age∗treatment) in the mixed effect models. To account for multiple testing, we applied the Benjamini-Hochberg procedure to control the false-discovery rate (FDR) for both the Model 1 and the Model 2. Statistical significance was set at a *p*-value <0.05. All statistical analyses were performed using SAS, version 9.4 (SAS Institute, Inc., Cary, North Carolina). The forest plots were produced in Graph Pad Prism, version 9.1 (Graph Pad Software, La Jolla, California).

## Results

### Cohort characteristics and supplementation

A total of 531 participants were screened of which 299 participants qualified and were randomized into the treatment groups. The demographics of the study population at Baseline are listed in Table 1, while those at Follow-up-1 and Follow-up-2 are listed in Table S2. There were no significant differences in participant characteristics between the placebo and carnosine arm at any visit. At Follow-up-1, 86 ​% of members of the carnosine supplementation group were compliant (>80 ​% of tablets consumed) while at Follow-up-2, 80 ​% of these participants were compliant. Only that data from cohort members who had greater than 80 ​% compliance for carnosine supplement use were used in the final analysis. We also excluded non-responders from the analysis, who were defined as those individuals in the carnosine group whose increase in urinary carnosine at Follow-up-1 or Follow-up-2 was <10 ​% of their baseline levels. Thus, we obtained useable cognitive testing results from 242 participants at Follow-up-1 and from 231 participants at Follow-up-2 (Fig. S2). As we recently reported [30], supplementation with carnosine was effective in increasing its basal levels in that randomized group, leading to an approximate two-fold increase in red blood cells levels, a seven-fold increase in urinary levels, and a two-fold increase in urinary levels of carnosine propanal. There were no significant differences in levels of urinary carnosine between the age group strata in samples obtained at any clinical visit (Table S3).

**Table 1:** Demographics of the randomized study population (Baseline).

Characteristics	Placebo (n ​= ​146)	Carnosine (n ​= ​153)	Total (n ​= ​299)
Age (yr), Mean ​± ​SD	44.8 ​± ​12.4	45.2 ​± ​12.3	45 ​± ​12.3
Sex - n (%)			
Male	62 (42.5 ​%)	64 (41.8 ​%)	126 (42.1 ​%)
Female	84 (57.5 ​%)	89 (58.2 ​%)	173 (57.9 ​%)
Race - n (%)			
White	115 (78.8 ​%)	127 (83 ​%)	242 (80.9 ​%)
Other	31 (21.2 ​%)	26 (17 ​%)	57 (19.1 ​%)
Income - n (%)			
< ​$45,000	36 (25.5 ​%)	41 (27.3 ​%)	77 (26.5 ​%)
$45,000-$89,999	52 (36.9 ​%)	51 (34.0 ​%)	103 (35.4 ​%)
>$90,000	53 (37.6 ​%)	58 (38.7 ​%)	111 (38.1 ​%)
Education - n (%)			
High school or less	6 (4.1 ​%)	7 (4.6 ​%)	13 (4.4 ​%)
Some college to 4- year degree	82 (56.2 ​%)	88 (57.5 ​%)	170 (56.9 ​%)
Masters or above	58 (39.7 ​%)	58 (37.9 ​%)	116 (38.8 ​%)

Age is presented as mean ​± ​standard deviation (SD) while other characteristics are presented as frequency (%). Age was calculated as the difference between the enrollment date and the date of birth and rounded to the nearest integer. Sex, race, income and education were self-reported.

### Carnosine supplementation and overall C*ognition* scores

Initially, we examined the impact of age and sex on overall speed, accuracy, and efficiency across all ten domains of the *Cognition* tests at Baseline. We found no difference in these outcomes between males and females or between the treatment groups (Table S4). However, these scores varied significantly with age. Scores for speed, accuracy, and efficiency were significantly higher for individuals 23–35 years of age than those in the older age groups (Fig. S3). The lowest scores were observed for those 51–65 years of age. This age-dependence is consistent with previous reports showing that age is strongly associated with cognitive ability [31,32].

**Fig. 1:**
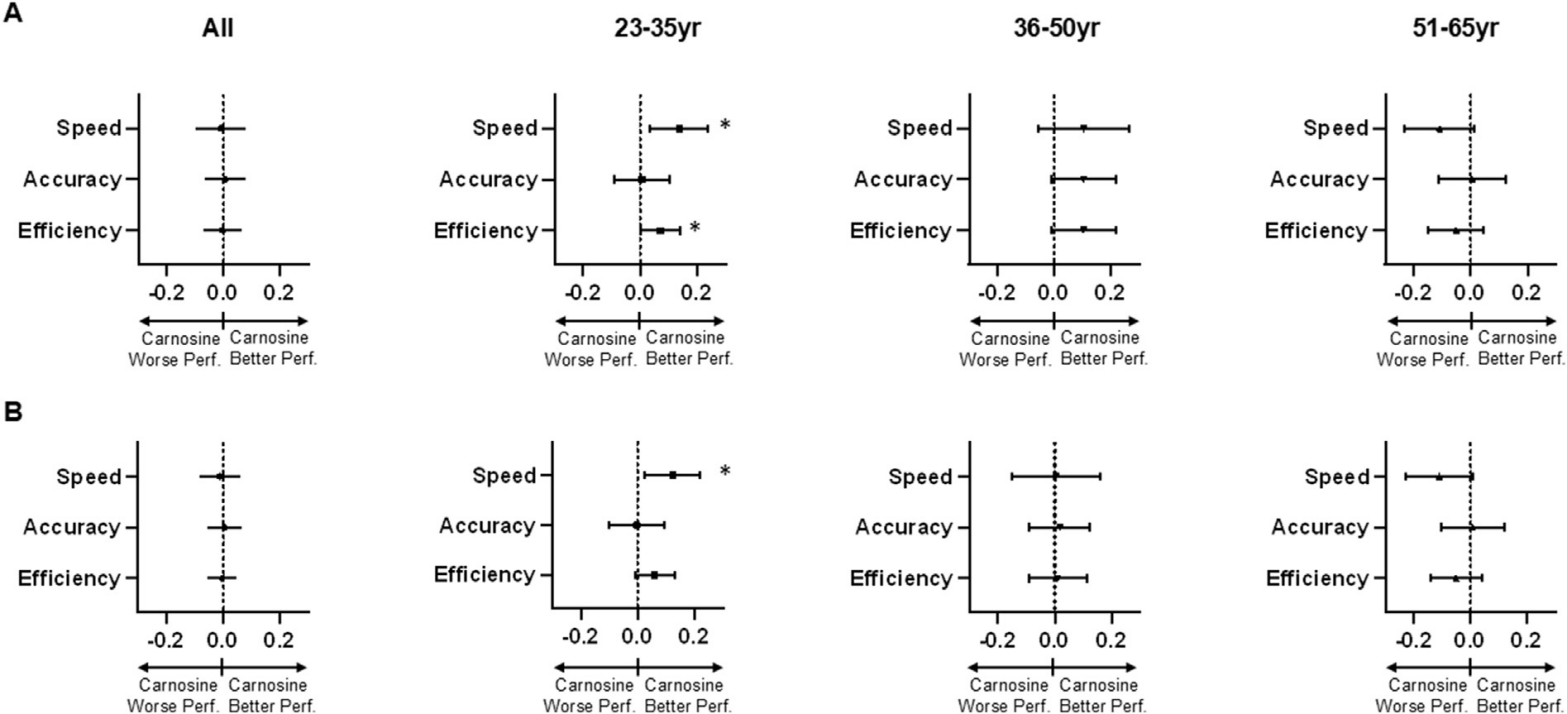
**Carnosine supplementation and overall standardized cognitive scores**. Illustrated are the regression coefficients for the effects of carnosine relative to placebo on overall speed, accuracy, and efficiency across all cognitive domains. This analysis was done for the total population (all) and for three stratified age groups (23–35yr: carnosine n ​= ​93, placebo n ​= ​129; 36–50yr: carnosine n ​= ​108, placebo n ​= ​135; 51–65yr: carnosine n ​= ​154, placebo n ​= ​153) as listed. The analysis was also done in an unadjusted model (A) and after adjustment for sex and race (B). ∗: adjusted p ​< ​0.05; error bars reflect 95 ​% confidence intervals; positive scores reflect higher speed/accuracy in the carnosine relative to the placebo group.

Next, we examined the effects of carnosine supplementation on cognition. When data from all participants were analyzed, either unadjusted or adjusted for age and sex, we found that there was no statistically significant difference in any scores between these groups (Fig. 1A and B). However, in the unadjusted model, both speed (0.138; 95 ​% CI: 0.037–0.238; p ​= ​0.007) and efficiency (0.072; 95 ​% CI: 0.000–0.143; p ​= ​0.0499) were significantly higher for participants 23–35 years of age in the carnosine arm versus the placebo arm (Fig. 1A) and, after adjusting for sex and race, speed (0.124; 95 ​% CI: 0.027–0.221; p ​= ​0.013), remained higher in this age group (Fig. 1B). No difference in speed, accuracy, and efficiency were observed between the carnosine and placebo arms in those in the older age groups in either model.

### Effect of carnosine supplementation on different cognitive domains

We further examined the effects of carnosine treatment on the speed and accuracy of the ten different domains measured in *Cognition* (see Table S1). At Baseline, in the whole study group, we found no statistical difference in any of these scores between the treatment groups (Table S5). However, we found that the accuracy value for the visual object learning test (VOLT), the speed and accuracy of the line orientation test (LOT) and the speed value for risk decision making (BART) were higher in males than in females (Table S6). On the other hand, the accuracy value of emotional recognition test (ERT) was higher in females than in males (Table S6). These sex differences are well established for the *Cognition* battery and cognitive testing in general [33]. In addition to sex, test scores at Baseline also varied with age. Of the 20 speed and accuracy scores, the lowest were observed for those in the oldest age group (51–65 years), while the youngest group had the highest scores (Table S6).

**Fig. 2:**
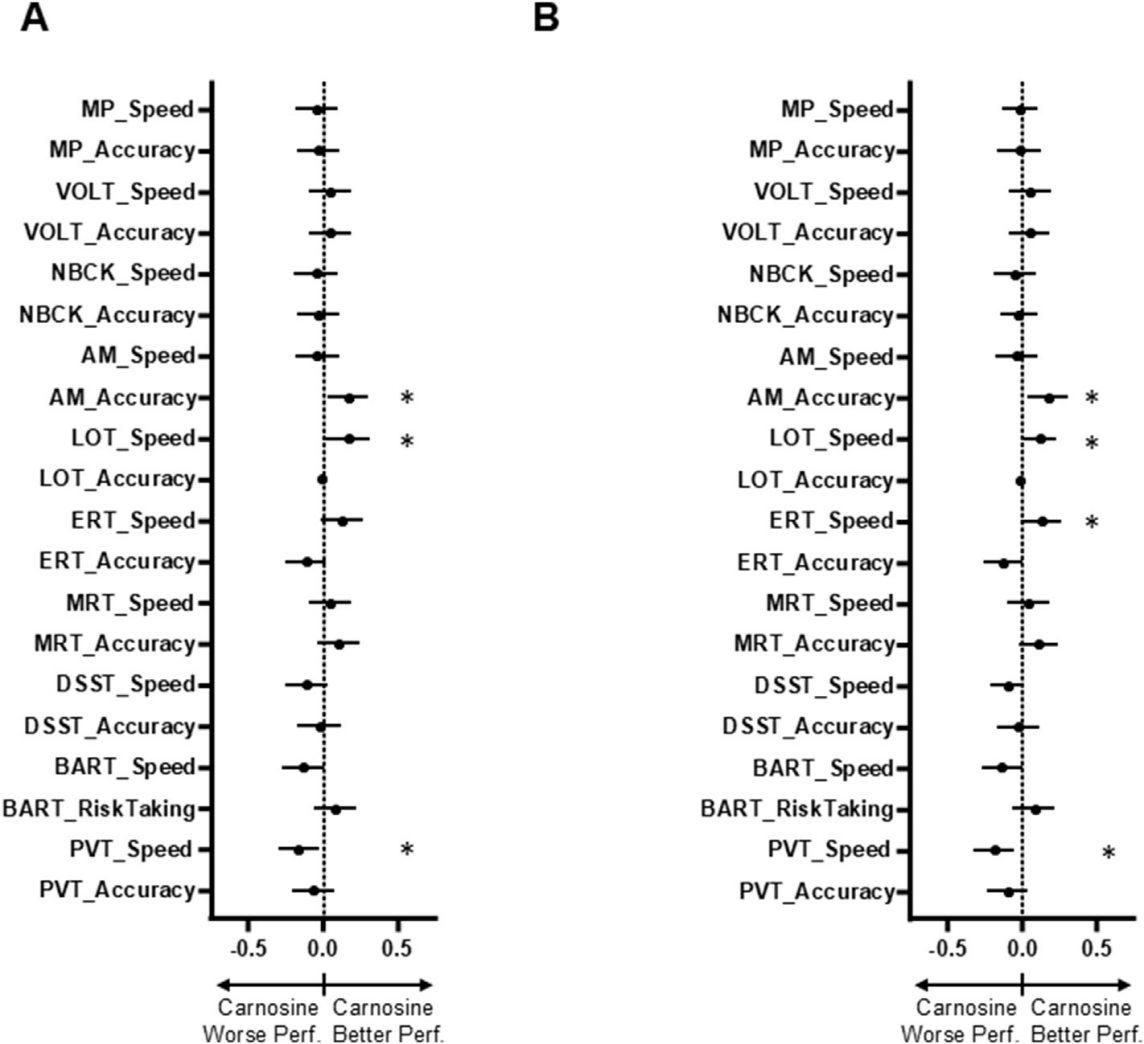
**Carnosine supplementation and individual standardized test scores for the whole population**. Illustrated are the regression coefficients for the effects of carnosine relative to placebo on speed and accuracy for each of the ten individual tests within the *Cognition* platform. This analysis was accomplished using a mixed effects model with no adjustments (Model 1: A) and after adjustment for age, sex, and race (Model 2:B). ∗: adjusted p ​< ​0.05; error bars reflect 95 ​% confidence intervals; positive scores reflect higher speed/accuracy in the carnosine relative to the placebo group; Abbreviations: MP: motor praxis; VOLT: visual object learning test; NBACK: fractal 2-back test; AM: abstract matching; LOT: line orientation; ERT: emotion recognition test; MRT: matrix reasoning test: DSST: digital symbol substitution test; BART: balloon analog risk task; PVT: psychomotor vigilance test.

After supplementation, several *Cognition* scores varied between the treatment groups (Fig. 2A). In an unadjusted mixed effects model, carnosine supplementation was associated with increases in the accuracy of the abstract matching test (AM; 0.170; 95 ​% CI: 0.033–0.308; p ​= ​0.015) and the speed value of the LOT (0.170; 95 ​% CI: 0.027–0.314, p ​= ​0.020). However, there was a decrease in the speed of the psychomotor vigilance test (PVT: 0.161; 95 ​% CI: −0.300 to −0.022; p ​= ​0.024). Similar changes were observed when the model was adjusted for race, sex, and age (Fig. 2B). Upon adjustment, the speed value of the ERT (0.135; 95 ​% CI: 0.002–0.268; p ​= ​0.046) was also increased in the carnosine supplementation group versus the placebo group.

**Fig. 3:**
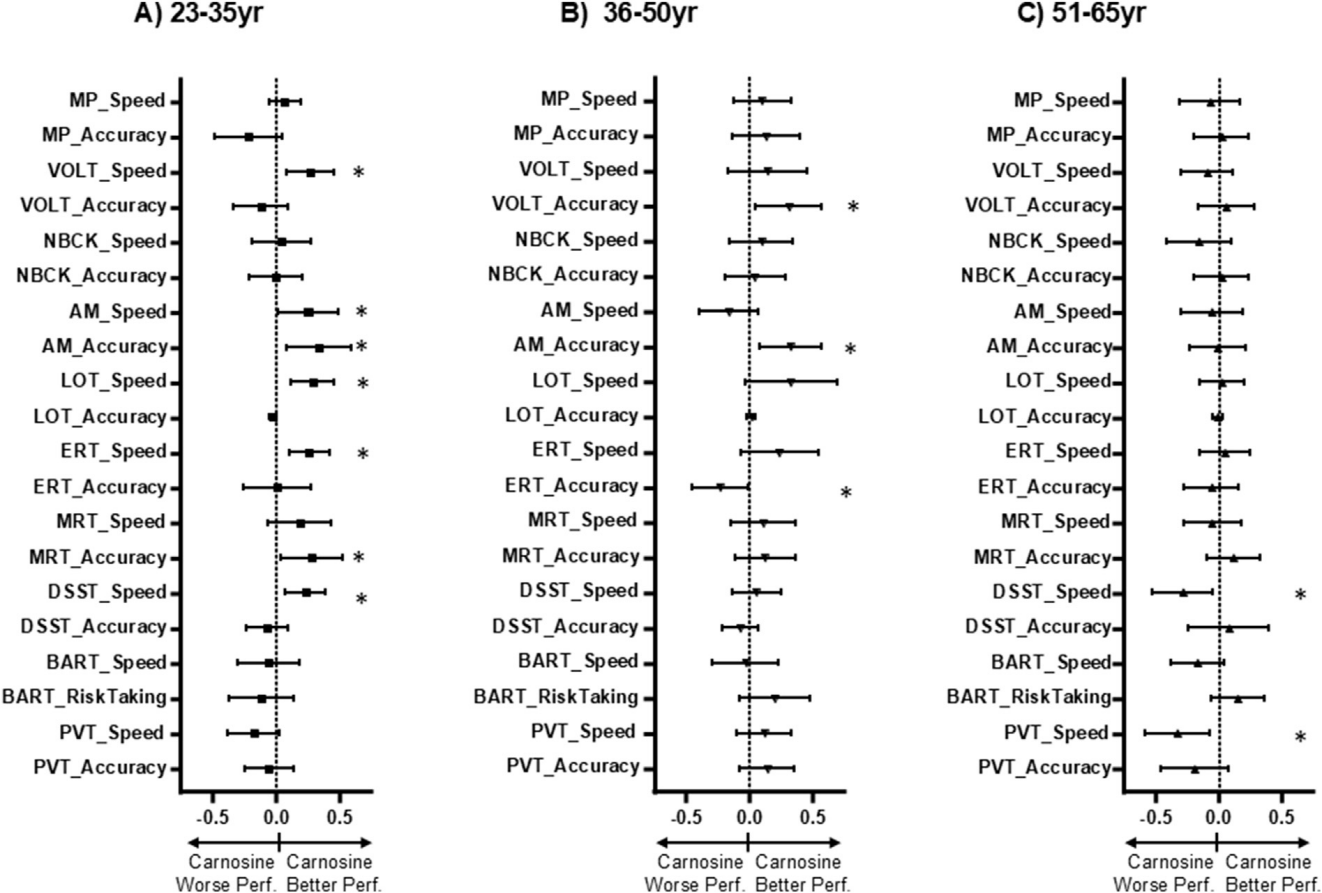
**Carnosine supplementation and individual test scores after age group stratification**. Illustrated are the regression coefficients for the effects of carnosine relative to placebo on speed and accuracy for each of the ten individual tests within the *Cognition* platform stratified by age (23–35yr: carnosine n ​= ​93, placebo n ​= ​129; 36–50yr: carnosine n ​= ​108, placebo n ​= ​135; 51–65yr: carnosine n ​= ​154, placebo n ​= ​153) using a mixed effects model with no adjustments. ∗: adjusted p ​< ​0.05; error bars reflect 95 ​% confidence intervals; positive scores reflect higher speed/accuracy in the carnosine relative to the placebo group; Abbreviations: MP: motor praxis; VOLT: visual object learning test; NBACK: fractal 2-back test; AM: abstract matching; LOT: line orientation; ERT: emotion recognition test; MRT: matrix reasoning test: DSST: digital symbol substitution test; BART: balloon analog risk task; PVT: psychomotor vigilance test.

The effects of carnosine were age-dependent (Fig. 3). In an unadjusted model, carnosine supplementation in those 23–35 years of age increased the speed value of the VOLT (0.268; 95 ​% CI: 0.080–0.457; p ​= ​0.005), the LOT (0.291; 95 ​% CI: 0.121–0.461; p ​= ​0.001), the ERT (0.266; 95 ​% CI: 0.110–0.421; p ​= ​0.001) and the digit symbol substitution test (DSST; 0.233; 95 ​% CI: 0.072–0.394; p ​= ​0.005). Also increased were the accuracy value of the matrix reasoning test (MRT; 0.282; 95 ​% CI: 0.036–0.528; p-0.025), and both speed and accuracy of the AM (0.255; 95 ​% CI: 0.017–0.492; p ​= ​0.036 and 0.336; 95 ​% CI: 0.082–0.591; p ​= ​0.010, respectively). For those between 36 and 50 years of age, carnosine supplementation led to improvements in accuracy for the VOLT (0.314; 95 ​% CI: 0.056–0.572; p ​= ​0.017) and the AM (0.329; 95 ​% CI: 0.088–0.571; p ​= ​0.008), but a decrease in the accuracy value of the ERT (−0.224, 95 ​% CI -0.445 to −0.003; p ​= ​0.047). No benefits of carnosine supplementation were seen with those 51–65 years of age. In fact, carnosine supplementation in this age group was associated with decreases in the speed of the DSST and PVT (DSST: −0.282, 95 ​% CI: −0.521 to −0.043; p ​= ​0.021; PVT: −0.324, 95 ​% CI: −0.583 to −0.065; p ​= ​0.014). The effects persisted after adjustment for sex and race (Fig. S4). The FDR-corrected p-values for this analysis are listed in Table S7.

**Fig. 4:**
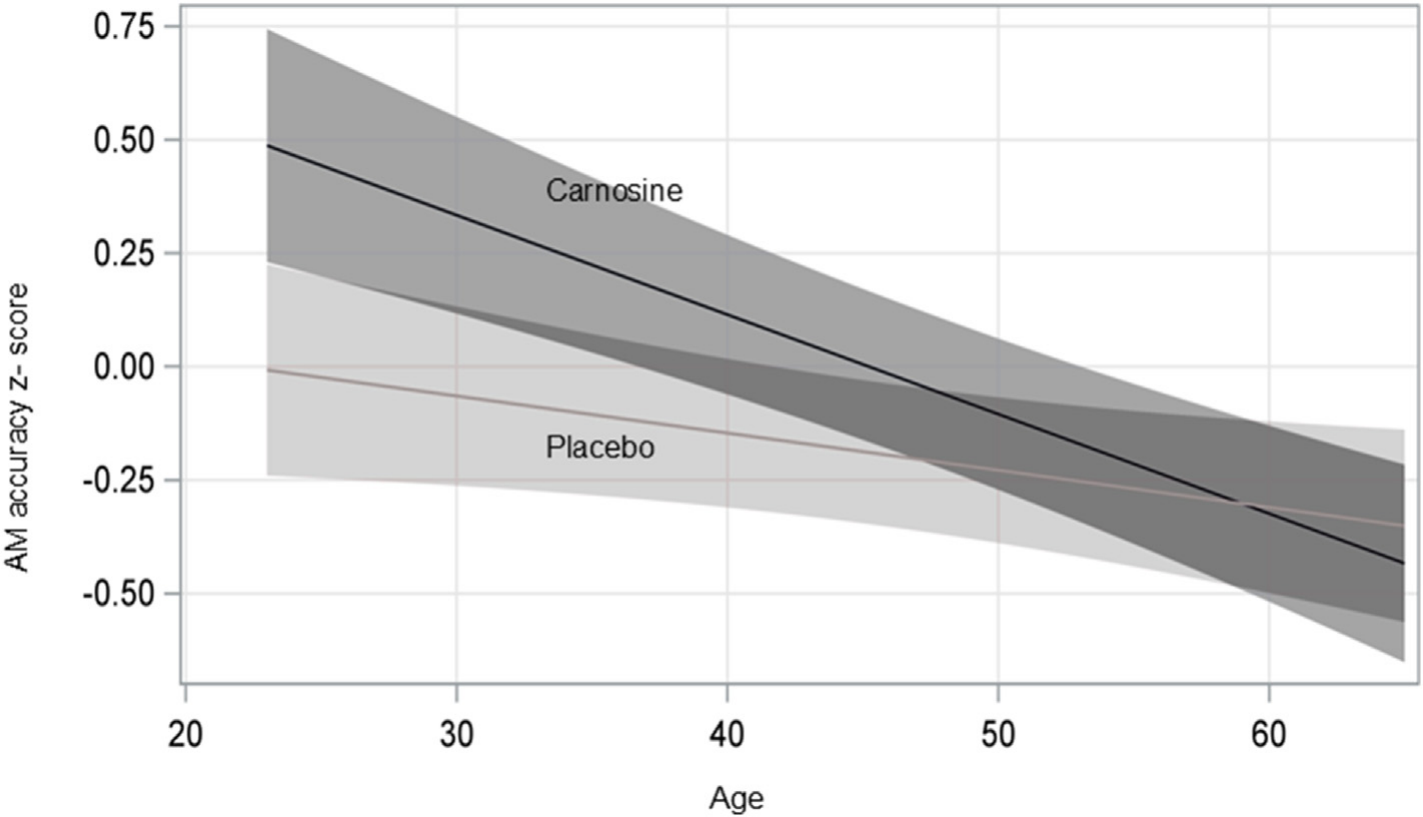
**Effect of supplementation and age on Abstract Matching accuracy**. A mixed effect model was used to examine the interaction between treatment (carnosine vs. placebo) and the numeric variable age. The model was adjusted for age, sex, race, and one interaction term treatment∗age. The variable visit was also included in the model to indicate the repeated measures of standardized Abstract Matching accuracy. The fit plot was computed at sex ​= ​Males, race ​= ​White and visit ​= ​4. The overall p value for the interaction term treatment∗age is 0.014 in this model.

Carnosine supplementation led to the largest improvements in accuracy for the AM in both the total study population (Fig. 2) and in the age-stratified groups (Fig. 3). The interaction between treatment (carnosine vs. placebo) and age was examined by including an interaction term (age∗treatment) in the mixed effect models where the AM accuracy score was the dependent variable. Using this analysis, we observed that the carnosine-mediated improvement was most apparent in younger individuals and its efficacy diminished with age (Fig. 4).

To determine if any potential bias might have been introduced because of the exclusion of non-compliant and non-responding participants in the carnosine supplementation group, we performed a sensitivity analysis in which all participants with cognition data were included. A similar level of improvement in the younger age group was observed in multiple individual tests using this intent to treat method.

## Discussion

Results from this work suggest that dietary carnosine supplementation improves cognitive function and that this improvement is most apparent in younger age groups. This improvement was observed in multiple individual tests which employ different cognitive domains and brain regions. Thus, in addition to the benefits reported for athletic performance [[34], [35], [36]], supplementary carnosine may have additional beneficial effects on cognition in young, healthy individuals.

While other studies have assessed the cognitive benefits of carnosine or β-alanine, these studies have been largely limited to older populations [37], using those under conditions of stress or extreme physical exertion [38,39], or in those with pre-existing conditions such as schizophrenia [17,40], Alzheimer's [16,41], multiple sclerosis [42], autism [43], or multi-symptom illness [44]. Results from these studies have been somewhat ambiguous with improvements in overall mood state or psychomotor behavior [38,39], but mixed benefits in cognitive testing scores [38,39,45,46]. Moreover, given that the goal of these prior studies was to determine whether supplementation could mitigate pre-existing cognitive deficiencies or disorders, they used instruments such as the Montreal cognitive assessment, global clinical dementia rating, or Wechsler memory scale (WMS), which measure mild cognitive decline or dementia.

In contrast, we studied the effects of carnosine supplementation in a cohort largely devoid of susceptibility factors or pre-existing conditions using the *Cognition* platform which is designed to measure performance in healthy adults [23]. The wide range of benefits we observed in younger individuals is consistent with the results of a related study showing that, in healthy young adults, the decline in verbal memory score (as measured by WMS) was significantly less in those consuming a carnosine-rich food product [47]. However, in that study, the benefits could not be linked to carnosine alone and no positive cognitive effects of the food were observed. In addition to human studies, some animal studies have also identified beneficial effects of carnosine or β-alanine intervention in reducing cognitive impairment and stress responses. However, these studies were likewise done using animal models of aging or disease [9,[11], [12], [13], [14],^48^,^49^].

We found only minimal effects of carnosine in a middle-aged population and none in older adults and reasons for this are not clear. While there were no significant differences in urinary carnosine levels between members of the age group strata at any clinical visit, we previously reported that supplementation with carnosine led to higher urinary levels of the carnosine-propanal conjugate in individuals who were >40 years of age than in those <40 years [30]. Given that this conjugate is derived from acrolein, which is produced by lipid peroxidation and inflammation [50,51], these observations suggest that in older individuals, carnosine is preferentially used to remove such products of oxidative stress and inflammation and, therefore, may be at insufficient levels for improving cognitive function. In contrast, younger individuals are likely to have lesser oxidative and inflammatory burden and/or more abundant antioxidant reserves and might not have to divert and exhaust carnosine stores for removing products of lipid peroxidation. However, such an explanation remains speculative and further studies are required to assess whether this is the case or whether greater amounts or a longer duration of carnosine supplementation may benefit older individuals as previously suggested [52]. Finally, the use of *Cognition* may have obscured the effects of carnosine on memory and retention as this platform is designed to measure high-level cognitive function rather than memory deficits or incipient dementia. Nevertheless, in *Cognition* tests, we did see an age-dependent decline in many domains, suggesting that the tests do measure age-dependent changes.

A major benefit of the *Cognition* platform is that performance on different tests could be mapped to different brain regions (Table S1). Therefore, improvements in test scores due to carnosine supplementation may provide some insights into its mechanism and site of action. Because carnosine acts as an intracellular buffer in improving muscle performance and facilitating glycolysis [3], it is likely to enhance brain function by the same means. The rates of aerobic glycolysis are higher in brain areas associated with high order cognitive function (prefrontal cortex, parietal lobe, cingulate cortex) than in the cerebellum and hippocampus [53]. The stronger benefits of carnosine on abstract matching, abstract reasoning, and paired associate learning, activities associated with prefrontal cortex, are consistent with the notion that carnosine may have selective benefits for those brain areas with high rates of glycolysis. Although such localization of carnosine action could not be achieved in our work, our results do offer a testable hypothesis that could verified in future direct neuroimaging studies. Furthermore, the rate of glycolysis decreases with age [54]. This age-dependent decrease in glycolysis may partly explain the inability of carnosine to improve cognitive function, as functional glycolytic activity may be required for carnosine to exert its effect.

In addition to facilitating glycolysis, carnosine may also impart protection because of its antioxidant properties. As a consequence of its high metabolic rate, the brain constantly produces free radicals [55]. Acting as a radical scavenger [3], carnosine may be able to limit downstream oxidative damage and inflammatory responses. Furthermore, carnosine can also chelate transition metals [3], which catalyze free radical production and protein aggregation. Finally, carnosine can permeate the blood brain barrier and stimulate glial cells to secrete neurotrophins such as brain-derived neurotrophic factor and nerve growth factor [56]. Indeed, β-alanine is thought to play a role as a neurotransmitter [57]. A comprehensive understanding of mechanisms underlying cognitive improvement requires further study.

Our study has several strengthens. It is the largest study on carnosine and cognition to date and it followed a rigorous double-blind, placebo-controlled study design. One limitation is the relatively small number of participants in the age group strata of the carnosine supplementation group, after exclusion of the non-responders and those who were non-compliant. Another limitation is the lack of dietary control as study participants consuming high levels of food rich in carnosine (e.g. poultry, red meat) may have higher endogenous levels independent of supplementation. However, the number of such individuals is likely to be small. Only a few individuals of the placebo group demonstrated abnormally high carnosine levels in follow-up visits, presumably due to meat consumption, and we assume similar numbers hold for those in the carnosine supplementation group. Because of randomization, it is unlikely that there are more high-level meat eaters in the carnosine supplementation group. Furthermore, absolute levels of carnosine typically obtained through diet (∼40 ​μM) [58] are smaller than levels measured in our carnosine supplementation group (∼86 ​μM). Thus we contend carnosine obtained from dietary sources makes only a minor contribution to overall levels in the carnosine supplementation group.

In summary, we found that daily supplementation with carnosine improved cognitive efficiency, mostly by improving speed without sacrificing accuracy, across a range of cognitive domains. These benefits were particularly pronounced in younger members of the study cohort (23–35 years) and were minimal or not observed in older study participants. Routine or targeted supplementation with carnosine may be an effective means of improving high-level cognitive performance in the young.

## Ethics approval

This study has Institutional approval (IRB #20.0258) and all participants signed an informed consent prior to involvement in the study.

## Data sharing

Data will be shared upon reasonable request to the communicating author.

## Author contributions

Concept and design: O'Toole, Amraotkar, Rai, Basner, Bhatnagar.

Acquisition, analysis, and interpretation of data: O'Toole, Amraotkar, Basner.

Drafting of the manuscript: all authors.

Critical review of the manuscript: all authors.

Statistical analysis: Gao, Sears, Rai.

Obtained funding: O'Toole, Bhatnagar.

Administrative, technical, or material support: O'Toole, Bhatnagar.

Supervision: O'Toole, Amraotkar.

## Funding support

This work was funded by grants (R01ES019217, P30ES030283) from the National Institutes of Health.

## Declaration of competing interest

The authors declare no conflicts of interest. The funder had no role in the design of the study or the decision to publish the results.
