# Supplementary material for: Carnosine supplementation improves cognitive outcomes in younger participants of the NEAT trial
Source: Neurotherapeutics. 2025 Feb 6;22(2):e00541. doi: 10.1016/j.neurot.2025.e00541 (PMC12014415; doi:10.1016/j.neurot.2025.e00541)

**Supplemental Material**

Table S1. Individual tests within the *Cognition* platform

Table S2. Demographics of the randomized study population (Follow-up-1 and Follow-up-2)

Table S3. Urinary carnosine levels.

Table S4. Overall Cognition scores at Baseline

Table S5. Individual cognition scores at Baseline

Table S6. Age- and sex-dependent changes on speed and accuracy scores of the individual tests of the Cognition platform

Table S7. FDR-corrected P values

Figure S1. Structure of carnosine.

Figure S2. Participant enrollment and *Cognition* measures

Figure S3. The association between age and *Cognition* scores at Baseline

Figure S4. Carnosine supplementation and individual test scores stratified by age group and adjusted for sex and race

| **Table S1. Individual tests within the *Cognition* platform** | | |
| --- | --- | --- |
| **Test** | **Cognitive domain assessed** | **Principal brain regions recruited** |
| Motor praxis (MP) | Sensory-motor speed | Sensorimotor cortex |
| Visual object learning (VOLT) | Spatial learning and memory | Medial temporal cortex, hippocampus |
| Fractal 2-back (F-2B) | Working memory | Prefrontal cortex, hippocampus |
| Abstract matching (AM) | Abstraction, concept formation | Prefrontal cortex |
| Line orientation (LOT) | Spatial orientation | Temporo-parietal cortex, visual cortex |
| Emotion recognition (ERT) | Emotion identification | Amygdala, hippocampus |
| Matrix reasoning (MRT) | Abstract reasoning | Prefrontal cortex, parietal cortex, temporal cortex |
| Digit symbol substitution (DSST) | Visual search, paired associate learning, working memory | Temporal cortex, motor cortex, prefrontal cortex |
| Balloon analog risk (BART) | Risk decision making | Orbital frontal cortex, amygdala, anterior cingulate cortex, ventral striatum |
| Psychomotor vigilance (PVT) | Attention | Prefrontal cortex, motor cortex, inferior parietal cortex |

| **Table S2. Demographics of the randomized study population (Follow-up-1 and Follow-up-2)** | | | | |
| --- | --- | --- | --- | --- |
| **Characteristics** | **Follow-up-1** | | **Follow-up-2** | |
|  | **Placebo (n=137)** | **Carnosine (n=105)** | **Placebo (n=134)** | **Carnosine (n=97)** |
| Age (yr), Mean ± SD | 44.8 ± 12.3 | 46.6 ± 12.6 | 44.7 ± 12.3 | 47.4 ± 12.1 |
| Sex - n (%) |  |  |  |  |
| Male | 60 (43.8%) | 42 (40%) | 57 (42.5%) | 46 (47.4%) |
| Female | 77 (56.2%) | 63 (60%) | 77 (57.5%) | 51 (52.6%) |
| Race - n (%) |  |  |  |  |
| White | 108 (78.8%) | 89 (84.8%) | 104 (77.6%) | 86 (88.7%) |
| Other | 29 (21.2%) | 16 (15.2%) | 30 (22.4%) | 11 (11.3%) |

Age is presented as a mean ± standard deviation (SD) while other characteristics

are presented as frequency (%). Sex and race were self-reported.

| **Table S3. Urinary carnosine levels** | | | |
| --- | --- | --- | --- |
| **Visit** | **Age Group (yr)** | **Mean (nm/mg creatinine)** | **SE** |
| **Randomization** | 23-35 | 9.16 | 1.56 |
|  | 36-50 | 10.3 | 2.02 |
|  | 51-65 | 8.21 | 1.49 |
| **Follow-up-1** | 23-35 | 80.1 | 24.0 |
|  | 36-50 | 84.1 | 17.3 |
|  | 51-65 | 99.7 | 16.8 |
| **Follow-up-2** | 23-35 | 89.1 | 21.6 |
|  | 36-50 | 75.9 | 18.1 |
|  | 51-65 | 97.5 | 15.1 |

Listed are the levels of urinary carnosine in participants of

the indicated age group strata at each clinical visit.

| **Table S4. Overall Cognition scores at Baseline** | | | | | | | |
| --- | --- | --- | --- | --- | --- | --- | --- |
|  |  | **Sex** | | | **Supplementation Group** | | |
| **Variable** | **Total (n=299)** | **Males (n=126)** | **Females (n=173)** | **P value** | **Placebo (n=146)** | **Carnosine (n=153)** | **P value** |
| Speed | -0.13 ± 0.79 | -0.12 ± 0.68 | -0.14 ± 0.71 | 0.765 | -0.14 ± 0.65 | -0.13 ± 0.74 | 0.861 |
| Accuracy | -0.004 ± 0.53 | 0.06 ± 0.58 | -0.05 ± 0.48 | 0.104 | 0.004 ± 0.53 | -0.01 ± 0.53 | 0.808 |
| Efficiency | -0.07 ± 0.53 | -0.03 ± 0.54 | -0.09 ± 0.51 | 0.310 | -0.14 ± 0.65 | -0.13 ± 0.74 | 0.861 |

Listed are the overall cognitive scores of the whole population, in the sex-stratified subgroups, and in the supplementation groups at baseline.

| **Table S5. Individual cognition scores at Baseline** | | | | |
| --- | --- | --- | --- | --- |
| **Test** | **Score** | **Placebo (n=146)** | **Carnosine (n=153)** | **P value** |
| MP | Speed | -0.15 ± 1.05 | -0.18 ± 1.06 | 0.816 |
|  | Accuracy | 0.09 ± 0.96 | -0.04 ± 1.07 | 0.265 |
| VOLT | Speed | 0.001 ± 0.96 | 0.04 ± 1.29 | 0.758 |
|  | Accuracy | -0.04 ± 0.88 | 0.02 ± 0.86 | 0.589 |
| NBACK | Speed | -0.14 ± 1.05 | -0.02 ± 1.11 | 0.363 |
|  | Accuracy | -0.10 ± 0.94 | -0.15 ± 0.96 | 0.626 |
| AM | Speed | -0.10 ± 1.15 | -0.11 ± 0.97 | 0.985 |
|  | Accuracy | 0.16 ± 0.59 | 0.19 ± 0.65 | 0.654 |
| LOT | Speed | -0.25 ± 1.69 | -0.05 ± 0.90 | 0.193 |
|  | Accuracy | 0.68 ± 0.17 | 0.67 ± 0.16 | 0.644 |
| ERT | Speed | -0.21 ± 1.22 | -0.03 ± 1.11 | 0.203 |
|  | Accuracy | 0.08 ± 0.92 | -0.003 ± 0.92 | 0.847 |
| MRT | Speed | -0.24 ± 1.01 | -0.17 ± 1.00 | 0.538 |
|  | Accuracy | 0.05 ± 1.04 | 0.27 ± 0.97 | 0.067 |
| DSST | Speed | -0.04 ± 1.00 | -0.06 ± 1.16 | 0.873 |
|  | Accuracy | -0.01 ± 1.69 | -0.01 ± 0.80 | 0.980 |
| BART | Speed | -0.17 ± 1.10 | -0.33 ± 1.28 | 0.262 |
|  | Risk Taking | 0.22 ± 1.12 | 0.27 ± 1.02 | 0.677 |
| PVT | Speed | -0.05 ± 1.16 | -0.28 ± 1.33 | 0.131 |
|  | Accuracy | -0.12 ± 1.14 | -0.29 ± 1.39 | 0.251 |

Listed are the speed and accuracy scores for each of the 10 individual *Cognitio*n tests

in the two supplement groups at Baseline. Abbreviations: MP: motor praxis; VOLT:

visual object learning test; NBACK: fractal 2-back test; AM: abstract matching; LOT:

line orientation; ERT: emotion recognition test; MRT: matrix reasoning test: DSST:

digital symbol substitution test; BART: balloon analog risk task; PVT: psychomotor

vigilance test.

| **Table S6. Age- and sex- dependent changes on speed and accuracy scores of the individual tests of the *Cognition* platform** | | | | | | | | | |
| --- | --- | --- | --- | --- | --- | --- | --- | --- | --- |
|  |  |  | **Age-dependence** | | | | **Sex-dependence** | | |
| **Test** | **Score** | **Total (n=299)** | **23-35yr (n=88)** | **36-50yr (n=97)** | **51-65yr (n=114)** | **p value** | **Males (n=126)** | **Females (n=173)** | **P value** |
| MP | Speed | -0.16 ± 1.05 | 0.54 ± 0.55 | -0.05 ± 0.90 | -0.80 ± 1.10 | <0.001 | -0.21 ± 1.04 | -0.13 ± 1.07 | 0.475 |
|  | Accuracy | 0.03 ± 1.02 | 0.19 ± 0.98 | -0.02 ± 1.11 | -0.06 ± 0.96 | 0.192 | 0.13 ± 1.07 | -0.05 ± 0.97 | 0.121 |
| VOLT | Speed | 0.02 ± 1.14 | 0.50 ± 0.68 | -0.14 ± 1.42 | -0.21 ± 1.04 | <0.001 | -0.04 ± 1.10 | 0.06 ± 1.17 | 0.460 |
|  | Accuracy | -0.01 ± 0.87 | 0.28 ± 0.81 | 0.04 ± 0.90 | -0.28 ± 0.83 | <0.001 | 0.14 ± 0.88 | -0.12 ± 0.85 | 0.009 |
| NBACK | Speed | -0.08 ± 1.08 | 0.21 ± 0.92 | 0.05 ± 0.96 | -0.45 ± 1.20 | <0.001 | -0.20 ± 1.12 | 0.01 ± 1.04 | 0.102 |
|  | Accuracy | -0.13 ± 0.95 | 0.38 ± 0.82 | -0.12 ± 0.90 | -0.51 ± 0.90 | <0.001 | -0.04 ± 0.95 | -0.19 ± 0.94 | 0.177 |
| AM | Speed | -0.11 ± 1.06 | 0.25 ± 1.06 | 0.09 ± 0.85 | -0.54 ± 1.06 | <0.001 | -0.12 ± 1.16 | -0.10 ± 0.98 | 0.855 |
|  | Accuracy | 0.17 ± 0.62 | 0.29 ± 0.63 | 0.24 ± 0.61 | 0.03 ± 0.60 | 0.004 | 0.15 ± 0.63 | 0.19 ± 0.62 | 0.541 |
| LOT | Speed | -0.15 ± 1.35 | 0.22 ± 0.72 | -0.20 ± 2.01 | -0.39 ± 0.88 | 0.006 | 0.09 ± 0.73 | -0.32 ± 1.64 | 0.008 |
|  | Accuracy | 0.68 ± 0.17 | 0.72 ± 0.13 | 0.70 ± 0.17 | 0.63 ± 0.18 | 0.001 | 0.71 ± 0.15 | 0.66 ± 0.18 | 0.007 |
| ERT | Speed | -0.12 ± 1.17 | 0.44 ± 0.66 | -0.20 ± 1.49 | -0.49 ± 0.99 | <0.001 | -0.18 ± 1.01 | -0.07 ± 1.27 | 0.401 |
|  | Accuracy | 0.01 ± 0.92 | 0.19 ± 0.92 | 0.06 ± 0.84 | -0.18 ± 0.96 | 0.015 | -0.12 ± 0.99 | 0.10 ± 0.85 | 0.041 |
| MRT | Speed | -0.21 ± 1.00 | 0.07 ± 0.91 | -0.22 ± 0.99 | -0.41 ± 1.04 | 0.004 | -0.30 ± 0.97 | -0.14 ± 1.02 | 0.162 |
|  | Accuracy | 0.16 ± 1.01 | 0.48 ± 0.98 | 0.37 ± 0.96 | -0.27 ± 0.93 | <0.001 | 0.26 ± 0.10 | 0.09 ± 1.01 | 0.153 |
| DSST | Speed | -0.05 ± 1.08 | 0.54 ± 0.74 | 0.19 ± 0.87 | -0.71 ± 1.13 | <0.001 | -0.09 ± 1.11 | -0.02 ± 1.07 | 0.531 |
|  | Accuracy | -0.01 ± 1.31 | 0.12 ± 0.57 | 0.11 ± 0.65 | -0.22 ± 1.97 | 0.104 | -0.06 ± 1.85 | 0.03 ± 0.70 | 0.573 |
| BART | Speed | -0.25 ± 1.20 | 0.25 ± 0.91 | -0.25 ± 1.28 | -0.64 ± 1.18 | <0.001 | -0.06 ± 1.25 | -0.39 ± 1.14 | 0.016 |
|  | Risk Taking | 0.25 ± 1.07 | 0.09 ± 1.03 | 0.34 ± 1.16 | 0.29 ± 0.10 | 0.249 | 0.30 ± 1.08 | 0.21 ± 1.06 | 0.477 |
| PVT | Speed | -0.17 ± 1.25 | 0.05 ± 0.94 | 0.03 ± 1.01 | -0.51 ± 1.55 | 0.001 | -0.03 ± 1.21 | -0.27 ± 1.27 | 0.102 |
|  | **Accuracy** | -0.21 ± 1.27 | 0.03 ± 0.95 | -0.07 ± 1.04 | -0.51 ± 1.58 | 0.004 | -0.12 ± 1.26 | -0.27 ± 1.28 | 0.293 |

Listed are the cognitive scores at Baseline in the total population and after stratification into three age group stratum and sex. Abbreviations: MP: motor praxis; VOLT: visual object learning test; NBACK: fractal 2-back test; AM: abstract matching; LOT: line orientation; ERT: emotion recognition test; MRT: matrix reasoning test: DSST: digital symbol substitution test; BART: balloon analog risk task; PVT: psychomotor vigilance test.

| **Table S7. FDR-corrected P values** | | | | | |
| --- | --- | --- | --- | --- | --- |
| **Test** | **Score** | **Model 1** | | **Model 2** | |
|  |  | **Raw P Value** | **FDR-corrected P value** | **Raw P Value** | **FDR-corrected P value** |
| MP | Speed | 0.249 | 0.415 | 0.215 | 0.359 |
|  | Accuracy | 0.110 | 0.244 | 0.112 | 0.248 |
| VOLT | Speed | **0.005** | **0.027** | **0.010** | **0.043** |
|  | Accuracy | 0.289 | 0.445 | 0.248 | 0.381 |
| NBACK | Speed | 0.721 | 0.801 | 0.976 | 0.976 |
|  | Accuracy | 0.992 | 0.992 | 0.788 | 0.875 |
| AM | Speed | **0.036** | 0.102 | **0.042** | 0.119 |
|  | Accuracy | **0.010** | **0.039** | **0.011** | **0.043** |
| LOT | Speed | **0.001** | **0.009** | **0.001** | **0.013** |
|  | Accuracy | 0.190 | 0.345 | 0.202 | 0.359 |
| ERT | Speed | **0.001** | **0.009** | **0.001** | **0.013** |
|  | Accuracy | 0.910 | 0.958 | 0.968 | 0.976 |
| MRT | Speed | 0.142 | 0.283 | 0.210 | 0.359 |
|  | Accuracy | **0.025** | 0.082 | **0.041** | 0.119 |
| DSST | Speed | **0.005** | **0.027** | **0.009** | **0.043** |
|  | Accuracy | 0.403 | 0.537 | 0.560 | 0.700 |
| BART | Speed | 0.619 | 0.729 | 0.618 | 0.727 |
|  | Risk Taking | 0.384 | 0.537 | 0.370 | 0.529 |
| PVT | Slowness | 0.095 | 0.238 | 0.098 | 0.246 |
|  | Accuracy | 0.580 | 0.725 | 0.432 | 0.576 |

Listed are the FDR-corrected p-values for individual tests of the Cognition battery using both

mixed effects models.


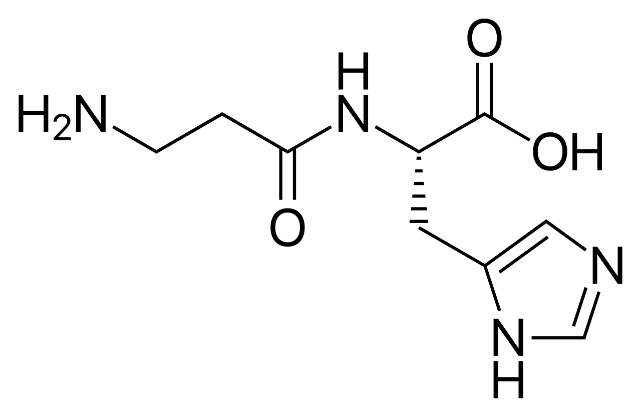


**Figure S1. Carnosine.** Illustrated is the chemical structure of carnosine.


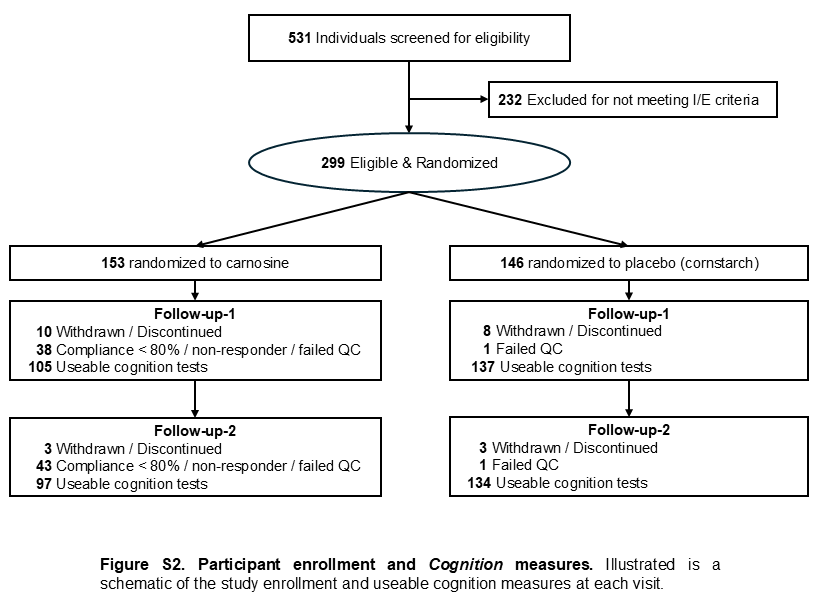


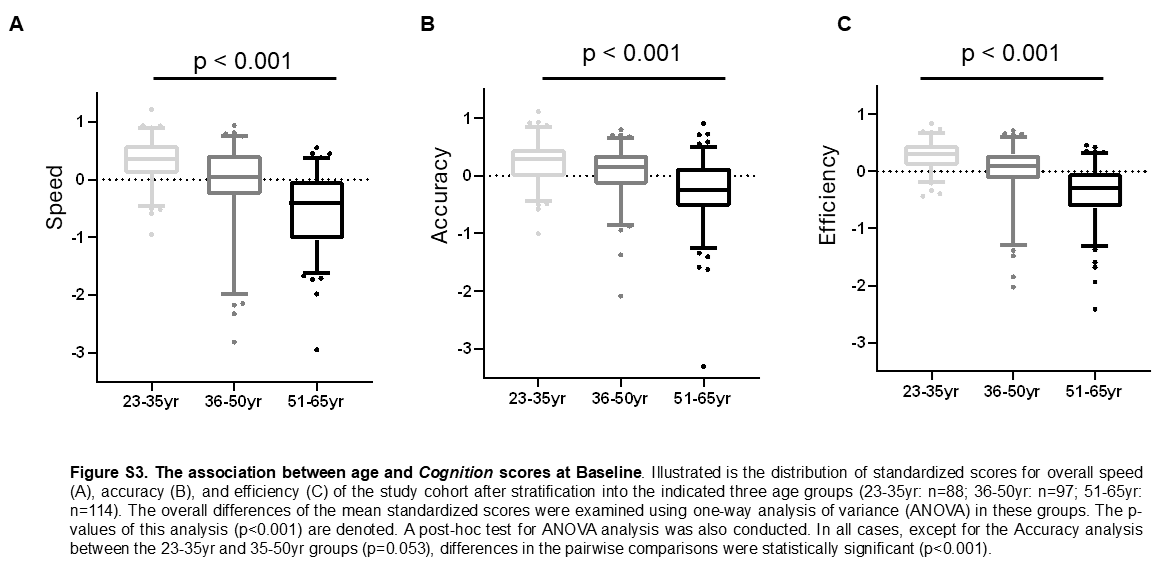


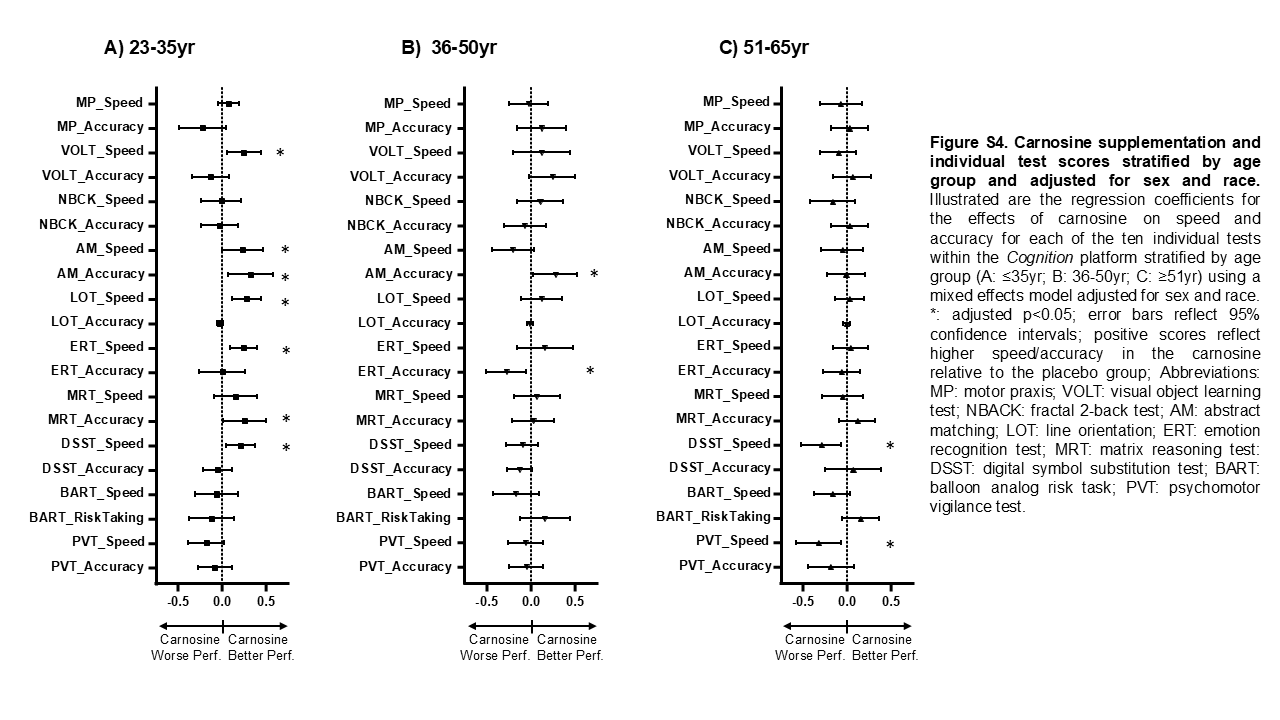

Supplement: Multimedia component 1 [file mmc1.docx]
